# Interactive Associations of Neuropsychiatry Inventory-Questionnaire Assessed Sleep Disturbance and Vascular Risk on Alzheimer’s Disease Stage Progression in Clinically Normal Older Adults

**DOI:** 10.3389/fnagi.2021.763264

**Published:** 2021-12-10

**Authors:** Omonigho M. Bubu, Ellita T. Williams, Ogie Q. Umasabor-Bubu, Sonya S. Kaur, Arlener D. Turner, Judite Blanc, Jaime Ramos Cejudo, Anna E. Mullins, Ankit Parekh, Korey Kam, Zainab T. Osakwe, Ann W. Nguyen, Antoine R. Trammell, Alfred K. Mbah, Mony de Leon, David M. Rapoport, Indu Ayappa, Gbenga Ogedegbe, Girardin Jean-Louis, Arjun V. Masurkar, Andrew W. Varga, Ricardo S. Osorio

**Affiliations:** ^1^Department of Psychiatry, Center for Sleep and Brain Health, NYU Grossman School of Medicine, New York, NY, United States; ^2^Department of Population Health, Center for Healthful Behavior Change, NYU Grossman School of Medicine, New York, NY, United States; ^3^Division of Epidemiology and Infection Control, State University New York (SUNY) Downstate Medical Center, Brooklyn, NY, United States; ^4^Evelyn F. McKnight Brain Institute, University of Miami Miller School of Medicine, Miami, FL, United States; ^5^Department of Psychiatry and Behavioral Sciences, Center for Translational Sleep and Circadian Sciences (TSCS), University of Miami Miller School of Medicine, Miami, FL, United States; ^6^Division of Pulmonary, Critical Care and Sleep Medicine at the Icahn School of Medicine at Mount Sinai, New York, NY, United States; ^7^College of Nursing and Public Health, Adelphi University, Garden City, NY, United States; ^8^Jack, Joseph and Morton Mandel School of Applied Social Sciences, Case Western Reserve University, Cleveland, OH, United States; ^9^Division of General Medicine and Geriatrics, Department of Medicine, Emory Brain Health Center, Emory University School of Medicine, Atlanta, GA, United States; ^10^Department of Epidemiology and Biostatistics, College of Public Health, University of South Florida, Tampa, FL, United States; ^11^Brain Health Imaging Institute, Department of Radiology, Weill Cornell Medicine, New York, NY, United States; ^12^Department of Neurology, Center for Cognitive Neurology, New York University School of Medicine, New York, NY, United States; ^13^Nathan S. Kline Institute for Psychiatric Research, Orangeburg, NY, United States

**Keywords:** sleep disturbance, Alzheimer’s disease, amnestic mild cognitive impairment, cardiovascular disease, biomarkers

## Abstract

**Background:** To determine whether sleep disturbance (SD) and vascular-risk interact to promote Alzheimer’s disease (AD) stage-progression in normal, community-dwelling older adults and evaluate their combined risk beyond that of established AD biomarkers.

**Methods:** Longitudinal data from the National Alzheimer’s Coordinating Center Uniform-Dataset. SD data (i.e., SD+ vs. SD-), as characterized by the Neuropsychiatric Inventory-Questionnaire, were derived from 10,600 participants at baseline, with at-least one follow-up visit. A subset (*n* = 361) had baseline cerebrospinal fluid (CSF) biomarkers and MRI data. The Framingham heart study general cardiovascular disease (FHS-CVD) risk-score was used to quantify vascular risk. Amnestic mild cognitive impairment (aMCI) diagnosis during follow-up characterized AD stage-progression. Logistic mixed-effects models with random intercept and slope examined the interaction of SD and vascular risk on prospective aMCI diagnosis.

**Results:** Of the 10,600 participants, 1,017 (9.6%) reported SD and 6,572 (62%) were female. The overall mean (SD) age was 70.5 (6.5), and follow-up time was 5.1 (2.7) years. SD and the FHS-CVD risk-score were each associated with incident aMCI (aOR: 1.42 and aOR: 2.11, *p* < 0.01 for both). The interaction of SD and FHS-CVD risk-score with time was significant (aOR: 2.87, *p* < 0.01), suggesting a synergistic effect. SD and FHS-CVD risk-score estimates remained significantly associated with incident aMCI even after adjusting for CSF (Aβ, T-tau, P-tau) and hippocampal volume (*n* = 361) (aOR: 2.55, *p* < 0.01), and approximated risk-estimates of each biomarker in the sample where data was available.

**Conclusions:** Clinical measures of sleep and vascular risk may complement current AD biomarkers in assessing risk of cognitive decline in older adults.

## Introduction

Mounting evidence recognizes Alzheimer’s disease (AD) as a multifactorial and heterogeneous disease with multiple contributors to its pathophysiology, including disturbed sleep (Bubu et al., 2017) and vascular risk factors (VRFs) (Gottesman et al., 2017). In cognitively normal individuals, disturbed sleep and VRFs have each been associated with brain Aβ deposition, tau aggregation, and neurodegeneration (Bangen et al., 2015; Branger et al., 2016; Lucey et al., 2019). All three are robust predictors and markers of future cognitive decline and the development of AD. However, identifying these biological markers requires the use of expensive and invasive methods like PET imaging and lumbar puncture for cerebrospinal fluid (CSF) collection. Therefore, it is imperative to improve the clinical characterization of the preclinical stages and identify additional risk factors and non-invasive, cost-effective surrogates of established AD biomarkers.

On the one hand and relevant to this study, our group and others have shown that self-reported sleep disturbances (SD) are associated with amyloid and tau pathology (Spira et al., 2013; Bubu et al., 2020). Objectively, changes in sleep characteristics, such as decreased non-rapid eye movement sleep (NREM) slow-wave activity (SWA), have been proposed as markers for amyloid and tau pathology (Winer et al., 2019). Poor sleep quality and sleep-EEG alterations also co-occur in mild cognitive impairment (MCI) and AD (Hita-Yanez et al., 2013). SDs can also predict a decline in simple measures of memory and global cognitive functioning in cognitively normal older adults at 1 year of follow-up (Potvin et al., 2012). Recently, we showed that reduced spindle density during NREM stage 2 sleep may represent an early dysfunction related to tau, possibly reflecting axonal damage or altered neuronal tau secretion (Kam et al., 2019). On the other hand, neuropathological studies indicate that vascular brain changes frequently co-occur with AD pathology and may lower the threshold for cognitive impairment (Esiri et al., 1999). Importantly, VRFs including hypertension are associated with lower brain glucose metabolism (Langbaum et al., 2012) and also higher Aβ(Langbaum et al., 2012; Gottesman et al., 2017) and tau burden (Langbaum et al., 2012; Gottesman et al., 2017). VRFs also act synergistically with Aβ burden to promote cognitive decline (Rabin et al., 2018). The combined impact of Aβ burden and cerebrovascular pathology has generally demonstrated additive effects on cognition (Marchant et al., 2013). Of significance to our study hypothesis is the fact that SD and VRFs commonly co-occur (Gottlieb et al., 2006; Gangwisch et al., 2007), as normal sleep significantly affects the cardiovascular system, with varying levels of autonomic regulation occurring during the different sleep stages (Legramante and Galante, 2005). Large epidemiologic studies also show self-reported SD associated with incident and prevalent VRFs including hypertension and diabetes (Gottlieb et al., 2006; Gangwisch et al., 2007). However, many patients with SD do not exhibit daytime symptoms and/or attribute sleep problems to cardiometabolic outcomes. Since multiple VRFs often coexist (Genest and Cohn, 1995) and incrementally increase the risk for AD (Luchsinger et al., 2005), the co-occurrence of VRFs and SD would be an important target for successful prevention strategies for AD. Furthermore, sleep and VRFs may serve as additional physiologic risks that may complement current (and future) biomarkers in assessing the risk of cognitive decline in older adults.

Using clinical setting data from a publicly available national repository, we examined whether SD was associated with prospective cognitive decline in a large cohort of cognitively normal older adults. We also examined time effects as it relates to the progression and conversion rates across SD groups. Second, we determined whether SD’s effect on prospective cognitive decline would be additive or synergistic with a well-validated, multivariable measure of vascular risk. Third, we evaluated the unique influence of the combined SD/VRFs effect beyond that of the commonly used AD biomarkers, including levels of CSF-Aβ, T-tau, P-tau, and hippocampal volume.

## Materials and Methods

### Study Design

Prospective longitudinal study utilizing data derived from the National Alzheimer’s Coordinating Center (NACC) uniform data set (UDS) covering the period from September 2005 to December 2018. NACC UDS is a data resource funded by the National Institute of Aging (NIA) and located at the University of Washington. The origin and development of the UDS are described elsewhere (Besser et al., 2018). Briefly, the UDS is a data repository containing deidentified clinical research data collected by the 33 NIA funded Alzheimer’s Disease Research Centers (ADRC). These deidentified data are made available to researchers by the NACC *via* a formal request to the NACC Steering Committee through the NACC website^1^. Each NACC individual site conducted its procedures in compliance with appropriate local laws, guidelines, and institutional review boards.

### Participants

Data were derived from 10,600 participants, aged between 65 and 99 years who were cognitively normal at baseline, with at least one completed UDS follow-up visit. Participants were required to have baseline medical data to quantify vascular risk using the Framingham Heart Study general cardiovascular disease (FHS-CVD) risk score. This group of participant data was used to examine the independent and combined effects of baseline SD and VRFs on prospective cognitive decline. A subset of the participants (*n* = 361) had baseline AD CSF biomarkers (i.e., CSF-Aβ, CSF P-tau, and CSF T-tau) and structural brain MRI data with at least one UDS follow-up visit. These individuals completed both lumbar puncture and MRI protocols. Data from this subset were used to evaluate the unique influence of the SD/FHS-CVD risk score combined risk on prospective cognitive decline beyond that of the established AD biomarkers.

### Sleep Disturbance

The NACC UDS repository lacks objective sleep measures, like polysomnography or actigraphy data, and other validated sleep questionnaires. NACC UDS only recently started collecting sleep disorders data. As such, the Neuropsychiatric Inventory Questionnaire (NPI-Q) (Cummings et al., 1994), which has been validated to evaluate psychopathology in dementia, was used to characterize SD. A trained health professional, *via* informant interview, administers the NPI-Q. Data includes an informant report, also covering severity, on the patient’s behaviors during the preceding month. Our focus was on the nighttime behavior disturbance item. Refer to Cummings et al. (1994) for specific wording of other NPI-Q items. Informant response to the question “Does the patient awaken you at night, rise too early in the morning, or take excessive naps during the day?” was categorized as positive (i.e., yes) or negative (i.e., no) for nighttime SD. Since this NPI-Q item characterizes nighttime behavioral disturbance and could potentially be conflated with other psychiatric conditions, especially if interpreted within the contexts of the participants’ possible neuropsychiatric/behavioral problems, we controlled for the possible effects of other NPI-Q reported psychiatric symptoms, such as hallucinations, delusions, apathy, and agitation in our analysis.

### CVD Risk

Vascular risk was quantified using the FHS-CVD risk score (D’Agostino et al., 2008), which provides a 10-year probability of future cardiovascular events (defined as coronary death, myocardial infarction, coronary insufficiency, angina, ischemic stroke, hemorrhagic stroke, transient ischemic attack, peripheral artery disease, and heart failure). Using baseline data, the FHS-CVD risk score was calculated. This score represents a weighted sum of age, sex, antihypertensive treatment (yes or no), systolic blood pressure (millimeters of mercury), body mass index, history of diabetes (yes or no), and current cigarette smoking status (yes or no). Higher scores represent a greater risk of cardiovascular events. In this sample, scores ranged from 5% to 89%. Similar to a previous study (Rabin et al., 2018), for stratified analyses and visualization purposes, participants were split into tertiles [at a FHSCVD risk score of 19% (lowest), 38% (middle), and 56% (highest)].

### CSF AD Biomarkers (CSF-Aβ, CSF-Tau, and CSF-PTau)

For this analysis, CSF values were available from a subset of clinically normal UDS participants (*n* = 361) from a small number of centers. ADRCs use different lab assay kits which provide ranges for amyloid beta and tau that are scaled completely differently for each brand of kit, have different ranges, and different cut-off values for positivity. Therefore, we utilized CSF data that were measured using the same assay method (i.e., ELISA) and standardized the data with center or batch-wise rescaling of the coefficient of variation from 20 to 10%, prior to combining the data for analyses. Site-by-site analyses limited the data and there was significant variation in the effect sizes though all in the same direction. Rescaling was performed using linear regression controlling for center-ID, age-at-baseline, sex, APOE4-status, and years-of-education with a reference batch. Very few participants have longitudinal CSF data; as such, NACC strongly cautioned using them for longitudinal analysis. CSF data used for this analysis were obtained only from baseline visits between September 2005 and October 2017.

### Magnetic Resonance Imaging (Hippocampal Volume)

Only a small subset of the clinically normal UDS participants had both structural MRI and CSF data available (*n* = 1,607); however, for this analysis, we excluded subjects whose CSF assay method was not ELISA and whose MRI data was not T1-weighted, leaving us with 361 subjects. Cortical reconstruction and volumetric segmentation were performed with the Freesurfer image analysis suite, which is documented and freely available for download online^2^. Data from this subset were used to evaluate the unique influence of the SD/FHS-CVD risk score combined risk on prospective cognitive decline beyond that of established AD biomarkers. Participants included in this analysis underwent high-resolution T1-weighted anatomical imaging to measure hippocampal volume. Hippocampal volumes were adjusted for total intracranial volumes by including total intracranial volumes as a covariate in the analytic models (Jack et al., 1989). Clinical UDS data were generally restricted to scans that were within ± 6 months from a UDS visit.

### Cognitive Normal and MCI Diagnosis

Participants at the ADRCs undergo detailed clinical and neuropsychological evaluation encompassing global cognitive functioning, processing speed, attention, working memory, executive functions, and episodic memory. Composite *Z*-scores are calculated for each cognitive domain, and a team of multidisciplinary clinicians (including neurologists and neuropsychologists) makes a clinical consensus diagnosis. Cognitive normal and MCI subjects scored between 24 and 30 on the mini-mental state examination (MMSE) whereas AD subjects scored between 20 and 26. Cognitive normal and MCI participants had global clinical dementia rating (CDR) scores of 0 and 0.5, respectively. At baseline, all participants had MMSE scores greater than 27, a CDR of 0, had ≤ 5 on the shorter version of the geriatric depression scale (Yesavage et al., 1982), and had a consensus clinical diagnosis of cognitively normal. For this analysis, our primary outcome was an incident amnestic MCI diagnosis *via* a consensus diagnosis using established clinical diagnostic criteria (McKhann et al., 1984) during UDS follow-up. As a secondary clinical outcome, we also investigated MCI as a global outcome (i.e., including both amnestic and non-amnestic MCI).

### Covariates/Potential Confounders

Covariates were selected *a priori* and included age, sex, BMI, education, and apolipoprotein E (APOE) ε4 status that was determined by the presence of at least one ε4 allele, clinical history of diabetes, hypertension, smoking, marital status, living arrangement, NPI-Q assessed comorbidity, and informant characteristics.

### Statistical Analyses

National Alzheimer’s Coordinating Center database has unbalanced data with an unequal number of measurements for each study participant. As such, our analyses included cox, multivariate analysis of covariance (MANCOVA), and multilevel mixed-effects regression models with normal errors (Harville, 1977; Laird and Ware, 1982; Jennrich and Schluchter, 1986) as they provide a flexible and valuable tool for analyzing such unbalanced longitudinal data. More importantly, they incorporate all the available information in the data and can reduce or even eliminate any bias resulting from an analysis confined to the complete cases (Ten Have et al., 1998).

Cox proportional hazards regression models estimated the effect of SD on the relative hazard of progression from being cognitively normal at baseline to an incident mild cognitive impairment (MCI)/amnestic mild cognitive impairment (aMCI) diagnosis at a UDS follow-up. Mean and median time to incident MCI/aMCI were also calculated. This analysis considered competing risks for death/attrition and controlled for age-at-baseline, sex, APOE4-status, years-of-education, clinical history of diabetes, hypertension, smoking, marital status, living arrangement, NPI-Q-assessed comorbidity (including other neuropsychiatric symptoms such as hallucinations, delusions, agitation, and apathy) and informant characteristics.

Multivariate ANOVA was conducted to test whether significant differences in mean conversion rates to aMCI existed between SD groups when the previous time-point was compared to the next. In the MANCOVA analysis, time was treated as discrete. To investigate the additive or synergistic associations of SD and cardiovascular burden with incident aMCI, logistic (i.e., non-linear) mixed-effects models with random intercept and slope were used to assess associations between SD, FHS-CVD risk score, and prospective cognitive decline, controlling for covariates/potential confounders, center-ID, and their interactions with time (operationalized as years from baseline for each participant). We examined interactions of SD with time and FHS-CVD risk score with time in a single model (model 1: incident aMCI (Yes vs. No) ≈ SD × time + FHS-CVD risk score × time + covariates × time). Next, we added an interaction term between SD, FHS-CVD risk score, and time to examine whether these two factors increase the likelihood of clinical diagnosis of incident aMCI at a UDS follow-up, beyond their separate effects (i.e., synergistic effect model: Incident aMCI (Yes vs. No) ≈ SD × FHS-CVD risk score × time + covariates × time). In the non-linear mixed-effects model, time was treated as continuous. We then conducted stratified analyses, splitting participants into tertiles based on FHSCVD risk score, comparing the risk of incident aMCI among the highest and middle tertile relative to the lowest tertile. Lastly, we examined the unique influence of the SD/FHS-CVD risk score combined risk on incident aMCI beyond that of hippocampal volume, CSF Aβ42, CSF Tau, and CSF PTau. We did this by evaluating the relative association of each biomarker with prospective cognitive decline by including all biomarkers within a single model (model: Incident aMCI (Yes vs. No) ≈ SD × FHS-CVD risk score × time + CSF-Aβ42 × time + hippocampal volume × time + CSF-Tau × time + CSF-PTau × time + covariates × time). For all analyses, we also tried to account for center differences statistical wise by including the center ID in the adjusted model. Statistical analyses were performed using SAS (version 9.4; SAS Institute Inc., Cary, NC, United States). For descriptive data *P*-values ≤0.05 were considered significant. For the MANCOVA, Cox models and mixed effects analyses, we controlled for family wise error and *P*-values ≤0.01, ≤0.017 and 0.025 were considered significant respectively.

### Data Availability

Deidentified or anonymized data are made available to researchers by the NACC *via* a formal request to the NACC Steering Committee through the NACC website^3^. Data from this study will be shared with qualified investigators upon request.

## Results

### Demographic and Clinical Characteristics

**FIGURE 1:**
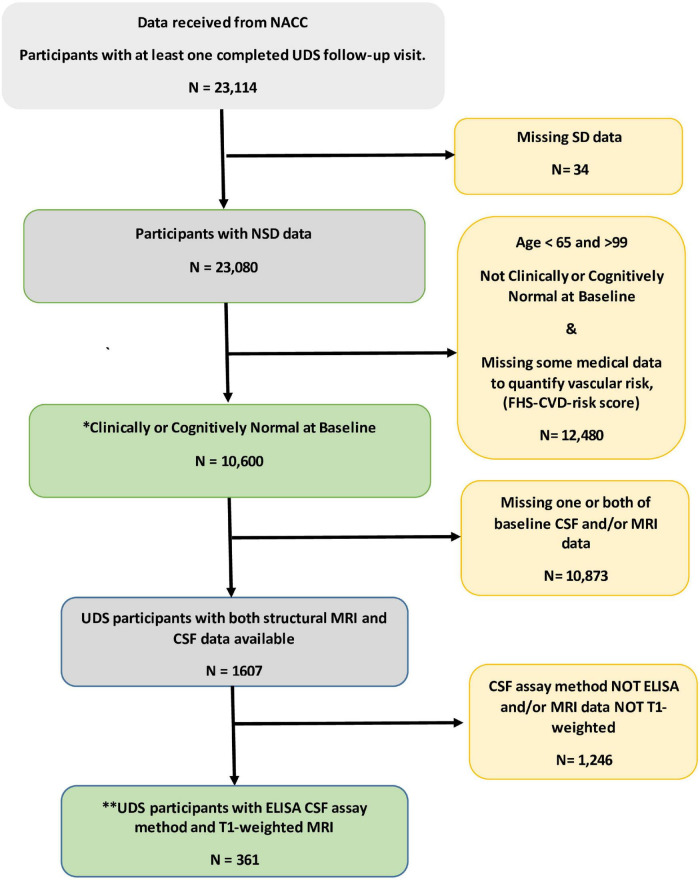
Flowchart for the selection of subjects in the study. * This group of participant data was used to examine independent and combined effects of sleep disturbance (SD) and vascular risk factors on prospect cognitive decline. ^**^Data from this subset were used to evaluate the unique influence of the SD/FHS-CVD risk score combined risk on prospective cognitive decline beyond that of established AD biomarkers. CSF, Cerebrospinal fluid; ELISA, enzyme linked immunosorbent assay; MRI, magnetic resonant imaging; NACC, National Alzheimer’s Coordinating Center; SD, sleep disturbance; UDS, uniform dataset.

Figure 1 shows the flowchart for the selection of subjects in the study. Table 1 shows the demographic and clinical characteristics of study participants at baseline. Of the 10,600 participants, 1,017 (9.6%) reported SD and were classified as SD+. The overall mean (SD) age was 75.5 (6.5). The mean (SD) ages of SD+ and SD- were 76 (7.3) and 75 (5.7) years, respectively. The overall mean (SD) follow-up time was 5.1 (2.7) years. The mean (SD) follow-up time was 5.2 (2.6) and 4.9 (2.7) for SD+ and SD- groups, respectively. Overall, female participants represented 62% of the sample with 60 and 62% representation in the SD+ and SD- groups, respectively. Participants in the SD groups did not significantly differ in age, ApoE4 status, BMI, and the FHS-CVD risk score. Of the 361 with AD biomarker data, 35 (9.7%) were SD+. In this group, SD- had significantly higher education and greater hippocampal volume relative to SD+. CSF amyloid and tau levels were similar for both SD groups.

**TABLE 1:** Baseline characteristics of cognitive normal study participants by NPI-Q-assessed sleep disturbance (SD), NACC UDS data.

Characteristics	All *n* = 10,600	Cognitive normal		*P*-value
		SD+ (*n* = 1,017)	SD- (*n* = 9,583)	
**Age**, mean (SD), y	75.5 (6.5)	76 (7.3)	75 (5.7)	0.14
**Average Follow-up time**, mean (SD), y	5.1 (2.7)	5.2 (2.6)	4.9 (2.7)	0.17
**Sex**				
Female sex, n ^*^(%)	6,572 (62)	611 (60)	5,961 (62)	< 0.01
Male sex, n ^*^(%)	4,028 (38)	406 (40)	3,622 (38)	
**Education**, years (SD)	16.0 (6.0)	15.5 (3.8)	16.5 (7.2)	< 0.01
**Modified FHS-CVD risk score**	32.8 (16.2)	33.8 (16.8)	31.7 (15.7)	0.11
Hypertension n ^*^(%)	167 (46)	18 (51)	149 (46)	0.04
Systolic Blood Pressure mm, Hg	141.5 (17.6)	142.8 (18.0)	139.8 (16.2)	0.33
Diabetes n ^*^(%)	1,166 (11)	142 (14)	1,024 (11)	0.44
Body mass index, mean ± SD	27.7 ± 4.8	27.9 ± 5.8	27.4 ± 5.0	0.23
Current smoker, n ^*^(%)	424 (4)	61 (6)	363 (4)	0.76
**Average number of vascular risk factors** (SD)	1.5 (1.1)	1.5 (1.3)	1.3 (1.1)	0.23
**Race/Ethnicity**				
Non-Hispanic White n ^*^(%)	8,162 (77)	793 (78)	7,369 (76.9)	< 0.0001
Non-Hispanic Black n ^*^(%)	1,474 (13.9)	87 (8.6)	1,387 (14.5)	
Hispanic n ^*^(%)	657 (6.2)	114 (11.2)	543 (5.6)	
Asian n ^*^(%)	307 (2.9)	22 (2.2)	285 (3.0)	
**Living Situation**				
Alone n ^*^(%)	3,434 (32.4)	301 (29.6)	3,133 (32.7)	0.14
With Others n ^*^(%)	7,166 (67.6)	716 (70.4)	6,450 (67.3)	
**Marital Status**				
Married/living with partner	6,455 (60.9)	646 (63.5)	5,809 (60.6)	0.23
Not currently married	3,424 (32.3)	307 (30.2)	3,117 (32.6)	
Never married/other	721 (6.8)	64 (6.3)	657 (6.9)	
**NPI-Q-assessed comorbidity**				
Hallucinations n ^*^(%)	12 (0.1)	2 (0.2)	10 (0.1)	0.51
Delusions n ^*^(%)	23 (0.2)	4 (0.4)	19 (0.2)	0.47
Agitation n ^*^(%)	51 (0.5)	3 (0.3)	48 (0.5)	0.43
Apathy n ^*^(%)	95 (0.9)	7 (0.7)	86 (0.9)	0.71
**Having at least one APOE ε4 allele**, n ^*^(%)	2,968 (28)	315 (31)	2,653 (28)	0.17
**MMSE** median (interquartile range)	29 (28, 30)	29 (27, 30)	29 (27, 30)	0.99
**CDR** median (interquartile range)	0 (0, 0)	0 (0, 0)	0 (0, 0)	0.99

**Characteristics**		**Cognitive Normal**		***P-value***
		**SD+ (%)**	**SD- (%)**	

**Informant characteristics at Baseline of the NACC UDS database by NPI-Q-assessed SD**				
**Relationship with patient**				
Spouse/partner		53.8	48.7	0.001
Non-spouse/partner		46.2	51.3	
**Living with patient**				
No		57.7	52.9	0.01
Yes		42.3	47.1	
**Sex**				
Female sex		61.9	62.8	0.63
Male sex		38.1	37.2	
**Question Reliability?**				
Yes		3.5	4.1	0.46
No		96.5	95.9	
CSF-ABETA mean (SE), pg/mL	374 (276.4)	372 (273.4)	378 (280.8)	0.86
CSFTAU mean (SE), pg/mL	402 (336.5)	412 (321.2)	393 (351.7)	0.79
CSFPTAU mean (SE), pg/mL	73 (21.6)	71 (24.6)	76 (18.2)	0.87
Hippocampal Volume Mean (SE), mm3	7,338 (724)	7,510 (767)	7,164 (662)	0.01

*(%), represents column percent; ABETA, Amyloid beta; CDR, Clinical Dementia Rating; CSF, Cerebrospinal fluid; FHS-CVD, Framingham heart study cardiovascular disease; MMSE, mini mental state examination; NACC UDS, National Alzheimer’s Coordinating Center Uniform Dataset; NPI-Q, neuropsychiatric inventory questionnaire; SD, sleep disturbance; PTAU, phosphorylated tau; SD, standard deviation. P-value ≤ 0.05.

### Association of NPI-Q Assessed SD and Prospective Cognitive Decline and Differences in Time Trend, Groups (SD+ vs. SD-), and Time-Points

One of the objectives of the analyses was to examine whether being clinically diagnosed as cognitively normal and having SD at baseline was associated with an incident aMCI, and secondarily a MCI (aMCI + non-amnestic MCI) diagnosis at a UDS follow-up. For the aMCI outcome, 195/1,017 (19%) SD positive subjects converted to aMCI relative to 958/9,583 (10%) SD negative subjects. For the general MCI (aMCI + non-amnestic MCI) outcome, 301/1,017 (30%) SD positive subjects converted to MCI relative to 1,607/9,583 (17%) SD negative subjects. We also wanted to determine time effects as it relates to progression and conversion rates across SD groups. Compared with SD- participants, SD+ participants had a significantly shorter time-to-progression to aMCI [mean ± SD (median) 4.2 ± 0.5 (4.0) years vs. 5.1 ± 0.4 (4.8) years, *p* < 0.001] and had an increased hazard risk of developing aMCI [adjusted hazard ratio (aHR): 2.49, 95% confidence interval (CI):1.18–3.81, *p* < 0.01] (Table 2A). Similar results were obtained with just an incident MCI (non-amnestic + amnestic) outcome (Table 2B). Compared with SD- participants, SD+ participants had a significantly shorter time-to-progression to MCI [mean ± SD (median) 4.0 ± 0.3 (3.8) years vs. 4.9 ± 0.4 (4.6) years, *p* < 0.001] and had an increased hazard risk of developing MCI [adjusted hazard ratio (aHR): 2.37, 95% confidence interval (CI):1.15–3.59, *p* < 0.01].

**TABLE 2:** Association of NPI-Q assessed sleep disturbance (SD) and an MCI diagnosis during follow-up in clinically normal older adults, NACC UDS data.

Characteristics N	aMCI n (%)	Mean time-to-aMCI	Median time-to-aMCI	^*^Model Estimate	*P*-value
		Years ± SD	Years ± SD	aHR 95% CI	
**(A) Association of NPI-Q Assessed Sleep Disturbance and an aMCI diagnosis during follow-up in Clinically Normal Older Adults, NACC UDS Data.**					
SD+ (*N* = 1,017)	195 (19)	4.2 (0.5)	4.0 (0.4)	2.49 (1.18, 3.81)	< 0.001
SD- (*N* = 9,583)	958 (10)	5.1 (0.4)	4.8 (0.5)	REF	

**Characteristics N**	**MCI n (%)**	**Mean time-to-MCI**	**Median time-to-MCI**	^*^Model Estimate	***P-value***
		**Years ± SD**	**Years ± SD**	**aHR 95% CI**	

**(B) Association of NPI-Q Assessed Sleep Disturbance and MCI (aMCI + non-amnestic MCI) diagnosis during follow-up in Clinically Normal Older Adults, NACC UDS Data**					
SD+ (*N* = 1,017)	301 (30)	4.0 (0.3)	3.8 (0.6)	2.37 (1.15, 3.59)	< 0.001
SD- (*N* = 9,583)	1,607 (17)	4.9 (0.4)	4.6 (0.2)	REF	

aHR, adjusted hazard ratio; aMCI, amnestic mild cognitive impairment; MCI, mild cognitive impairment; NACC UDS, National Alzheimer’s Coordinating Center Uniform Dataset; SD, standard deviation; *Cox model effect estimate, adjusted for age-at-baseline, sex, APOE4-status, years-of-education, clinical history of diabetes, hypertension, smoking, marital status, living arrangement, NPI-Q assessed co-morbidity and informant characteristicsand center-ID. P-value = 0.05/2 ≤ 0.025 controlling for family wise error.

Table 3 shows MANCOVA results testing the differences in the mean incident aMCI conversion rates over time, based on SD status. The Pillai’s trace test values presented examine time-point*SD effect and provide the exact *F* statistics for the time trend of mean incident aMCI conversion rates across SD groups. The time-point effect provides the exact F statistics for the mean incident aMCI conversion rates over time. Other statistical interpretations are delineated in the Table 3 footnote. The data showed that the time trend of incident aMCI conversion rates differed across SD groups (Pillai’s trace test, *p* < 0.01 for all). Across all subjects, mean incident aMCI conversion rates increased significantly over time (Pillai’s trace test, *p* < 0.001 for all). For the comparisons of repeated measures, there were significant differences in mean incident aMCI conversion rates across the SD groups, with SD+ generally having higher conversion rates than SD-, when the previous time-point was compared with the next (*p* ≤ 0.01 for all).

**TABLE 3:** Multivariate ANCOVA results testing mean aMCI change conversion rates in time trend, groups and time points, NACC UDS data.

MANCOVA Test Criteria	Statistic	Value	F-value	*P*-value
**Mean conversion rates to aMCI**				
**All Participants**				
Timepoint*SD Effect	Pillai’s Trace	0.235	6.46	0.0023*
Timepoint Effect	Pillai’s Trace	0.662	43.66	< 0.0001*
**Repeated Measures Analysis of Covariance of Contrast Variables SD+ vs. SD-**				
timepoint_1 (Year 2)			0.14	0.7125
timepoint_2 (Year 3)			4.93	0.0031*
timepoint_3 (Year 4)			7.58	0.0031*
timepoint_4 (Year 5)			8.82	0.0031*
timepoint_1 vs. timepoint_0			6.51	0.0113*
timepoint_2 vs. timepoint_1			34.22	< 0.0001*
timepoint_3 vs. timepoint_2			23.57	< 0.0001*
timepoint_4 vs. timepoint_3			13.12	0.0009*
timepoint_5 vs. timepoint_4			8.08	0.0006*

MANCOVA, Multivariate Analysis of Covariance. aMCI, amnestic mild cognitive impairment; SD, sleep disturbance; MANCOVA test criteria interpretations, Timepoint*DS Effect provides the exact F statistics for the time trend of mean incident aMCI conversion rates across SD groups. The time-point effect provides the exact F statistics for the mean incident aMCI conversion rates over time. Repeated Measures Analysis of Variance of Contrast Variables SD+ vs. SD- interpretations: Timepoint_1 (Year 2) provides the F statistic for the difference in mean incident aMCI conversion rates across SD groups over time from baseline (Year 1) to Year 2; Timepoint_2 (Year 3) provides the F statistic for the difference in mean incident aMCI conversion rates across SD groups over time from baseline (Year 1) to Year 3 etc.; timepoint_1 vs. timepoint_0 provides the F statistic for the difference in mean incident aMCI conversion rates across SD groups over time when timepoint 1 (Year 2) is compared to baseline (Year 1); timepoint_2 vs. timepoint_1 provides the F statistic for the difference in mean incident aMCI conversion rates across SD groups over time when timepoint 2 (Year 3) is compared to timepoint 1 (Year 2) etc. *P-value = 0.05/5 = ≤0.01controlling for family wise error.

### Interactive Associations of NPI-Q Assessed SD and Vascular Risk With Prospective Cognitive Decline

A second objective was to determine whether the presence of SD at baseline and an elevated FHS-CVD risk score were additive or synergistic in their associations with an incident aMCI diagnosis at a UDS follow-up. We examined interactions of SD with time and FHS-CVD risk score with time in a single model (i.e., Model 1, Table 4). Both SD and having a higher FHS-CVD risk score were associated with an incident aMCI diagnosis at a UDS follow-up. Since age and sex are incorporated into the FHS-CVD risk score, we conducted sensitivity analyses omitting age and sex as covariates and these yielded similar results (e.g., SD’s significant effect on incident aMCI changed from aOR:1.42 to 1.55, *p* < 0.003). The model (i.e., Model 2, Table 4) that included the interaction between SD and FHS-CVD risk score (i.e., the synergistic effect model) yielded a significant interaction term suggesting that SD and an elevated FHS-CVD risk score together increased the likelihood of having an incident aMCI at a UDS follow-up beyond their individual effects. For strata specific effects, we stratified FHS-CVD risk score into tertiles. Participants with SD and in the highest and middle tertiles of the FHS-CVD risk score were significantly more likely to develop incident aMCI during UDS follow-up, compared with participants without SD in the lowest FHS-CVD risk score tertile (OR: 2.82, 95%CI, 1.23–4.35; *p* < 0.003 and OR: 2.38, 95%CI 1.17–3.59; *p* < 0.001, respectively; as shown in Model 2, Table 4). Overall, the findings were similar for the MCI (non-amnestic + amnestic) outcome; however, with attenuated but significant effects seen for SD and stronger effects seen for FHS-CVD risk and the interaction between SD and FHS-CVD risk score (i.e., the synergistic effect model, as shown in Models 1 and 2, Table 5).

**TABLE 4:** Interactive associations of NPI-Q assessed sleep disturbance (SD) and vascular risk with an aMCI diagnosis during follow-up in clinically normal older adults, NACC UDS data.

Outcome	^*^Model 1 Term	^**^aOR (95% CI)	^***^Pvalue
Conversion Risk from CN to aMCI	SD^*^time	1.42 (1.15–1.71)	< 0.003
	FHS-CVD*time	2.11 (1.18–3.04)	< 0.001

**Outcome**	^*^Model 2 Term	**aOR (95% CI)**	***P*value**

Conversion Risk from CN to aMCI	FHS-CVD^*^SD^*^time	2.87 (1.18–4.56)	< 0.001
^*^^#^Model 2 Term FHS-CVD Stratified Analyses (SD+ vs. SD-)			
Conversion Risk from CN to aMCI	Highest FHS-CVD tertile	2.82 (1.23–4.35)	< 0.003
	Middle FHS-CVD tertile	2.38 (1.17, 3.59)	< 0.001
	Lowest FHS-CVD tertile	REF	REF

**Outcome**	^*^Model 3 Term	**aOR (95% CI)**	***P*value**

Conversion Risk from CN to aMCI	SD^*^time	1.28 (1.00–1.57)	0.057
	FHS-CVD^*^time	1.63 (1.22–2.07)	< 0.003
	FHS-CVD^*^SD^*^time	2.55 (1.14–3.96)	< 0.001
	CSF-Aβ^*^time	3.03 (1.78–4.45)	< 0.001
	CSF-Tau^*^time	3.37 (1.65–5.09)	< 0.001
	CSF-PTau^*^time	3.61 (1.72–5.51)	< 0.001
	Hippocampal Volume^*^time	2.13 (1.36–2.85)	< 0.005

aMCI, amnestic mild cognitive impairment; FHS-CVD, Framingham heart study cardiovascular disease; NACC UDS, National Alzheimer’s Coordinating Center Uniform Dataset; SD, sleep disturbance; 95%CI, 95% confidence interval; *Model term assessed, **Model Adjusted for age, sex, BMI, education, ApoE4 status, clinical history of diabetes, hypertension, smoking, marital status, living arrangement, NPI-Q assessed co-morbidity and informant characteristicsand center-ID; *^#^Model 2 Term FHS-CVD Stratified Analyses (SD+ vs. SD-). The FHS-CVD Stratified Analyses (SD+ vs. SD- corresponds to Model 2 where we investigated the FHS-CVD*SD*time interaction term. Since SD is a categorical variable, using data driven techniques we split the FHS-CVD risk score into tertiles within the SD groups. This was done for stratified analyses and for visualization purposes to generate strata specific estimates. **aOR, adjusted odds ratios obtained for logistic mixed effect model beta estimates. ***P-value = 0.05/3 ≤0.017 controlling for family wise error.

**TABLE 5:** Interactive associations of NPI-Q assessed sleep disturbance (SD) and vascular risk with an MCI (aMCI + non-amnestic MCI) diagnosis during follow-up in clinically normal older adults, NACC UDS data.

Outcome	^*^Model 1 Term	^**^aOR (95% CI)	^***^Pvalue
Conversion Risk from CN to MCI	SD^*^time	1.37 (1.10–1.67)	< 0.007
	FHS-CVD^*^time	3.24 (1.72–4.76)	< 0.001

**Outcome**	^*^Model 2 Term	**aOR (95% CI)**	***P*value**

Conversion Risk from CN to aMCI	FHS-CVD^*^SD^*^time	3.95 (2.18–5.71)	< 0.001
^*^^#^Model 2 Term FHS-CVD Stratified Analyses (SD+ vs. SD-)			
Conversion Risk from CN to aMCI	Highest FHS-CVD tertile	3.87 (2.23–5.51)	< 0.003
	Middle FHS-CVD tertile	2.88 (1.47, 4.29)	< 0.001
	Lowest FHS-CVD tertile	REF	REF

**Outcome**	^*^Model 3 Term	**aOR (95% CI)**	***P*value**

Conversion Risk from CN to aMCI	SD^*^time	1.22 (1.03–1.41)	0.043
	FHS-CVD^*^time	2.67 (1.22–4.12)	< 0.003
	FHS-CVD^*^SD^*^time	2.78 (1.29–4.38)	< 0.001
	CSF-Aβ^*^time	2.89 (1.43–4.35)	< 0.001
	CSF-Tau^*^time	4.47 (2.65–6.29)	< 0.001
	CSF-PTau^*^time	3.01 (1.12–4.91)	< 0.001
	Hippocampal Volume^*^time	2.52 (1.37–3.67)	< 0.005

MCI, mild cognitive impairment; FHS-CVD, Framingham heart study cardiovascular disease; NACC UDS, National Alzheimer’s Coordinating Center Uniform Dataset; SD, sleep disturbance; 95%CI, 95% confidence interval; *Model term assessed, **Model Adjusted for age, sex, BMI, education, ApoE4 status, clinical history of diabetes, hypertension, smoking, marital status, living arrangement, NPI-Q assessed co-morbidity and informant characteristicsand center-ID; *^#^Model 2 Term FHS-CVD Stratified Analyses (SD+ vs. SD-). The FHS-CVD Stratified Analyses (SD+ vs. SD- corresponds to Model 2 where we investigated the FHS-CVD*SD*time interaction term. Since SD is a categorical variable, using data driven techniques we split the FHS-CVD risk score into tertiles within the SD groups. This was done for stratified analyses and for visualization purposes to generate strata specific estimates. **aOR: adjusted odds ratios obtained for logistic mixed effect model beta estimates. ***P-value = 0.05/3 ≤0.017 controlling for family wise error.

### Associations of NPI-Q-Assessed SD and Vascular Risk With Prospective Cognitive Decline Controlling for CSF AD Biomarkers (CSF-Aβ, CSF-Tau, and CSF-Ptau) and Hippocampal Volume

Our third objective was to evaluate the unique influence of the SD/FHS-CVD risk score combined risk on an incident aMCI diagnosis at a UDS follow-up beyond that of commonly used AD biomarkers, including levels of CSF-Aβ, CSF-Tau, and CSF-PTau and hippocampal volume. We did this by evaluating the relative association of each biomarker with an incident MCI diagnosis at a UDS follow-up by including all biomarkers within a single model (Model 3, Table 4). SD’s effect on an incident aMCI diagnosis at a UDS follow-up was attenuated (aOR: 1.28, 95%CI, 1.00–1.57; *p* = 0.057) when all AD biomarkers were included in the model.

**FIGURE 2:**
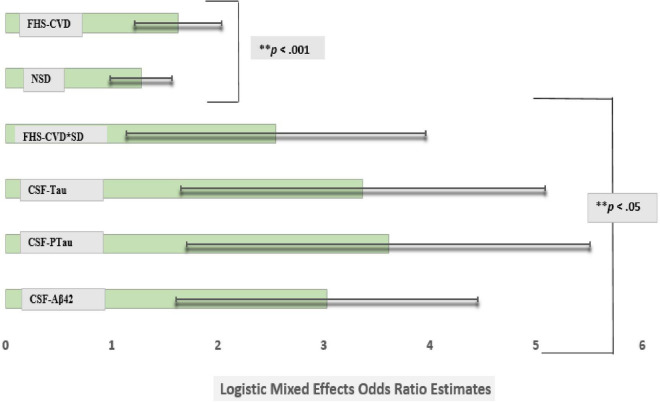
Comparison of logistic mixed effects model estimates of CSF Aβ42, CSF Tau, CSF PTau, SD, and vascular risk on the risk of conversion from cognitively normal to amnestic mild cognitive impairment (MCI). Sleep disturbance’s (SD) effect on an incident amnestic MCI diagnosis at a UDS follow-up was attenuated after adjusting for commonly used Alzheimer’s disease markers in the same model (model 3). However, the Framingham Heart Study-general cardiovascular disease (FHS-CVD) risk score and the interaction between SD and FHS-CVD risk score remained significantly associated with an incident MCI diagnosis at a uniform dataset (UDS) follow-up even after including these molecular biomarkers in the model. More importantly, the SD and FHS-CVD risk score synergism approximated the risk estimates of each molecular AD biomarker though significantly different. ^**^Multiple comparisons: FHS-CVD*SD vs. CSF-Aβ42, FHS-CVD*SD vs. CSF-Tau and FHS-CVD*SD vs. CSF-PTau (*p* < 0.01 for all).

However, the FHS-CVD risk score and the interaction between SD and FHS-CVD risk score remained significantly associated with an incident aMCI diagnosis at a UDS follow-up even after including these AD biomarkers in the model (OR: 1.63, 95%CI, 1.22–2.07; *p* < 0.003 and OR: 2.55, 95%CI, 1.14–3.96; *p* < 0.001, respectively). Overall, the findings were similar for the MCI (non-amnestic + amnestic) outcome, as shown in Model 3, Table 5. More importantly, the SD and FHS-CVD risk score synergism approximated the risk estimates of each molecular AD biomarker, even though they were significantly different (Figure 2).

## Discussion

We examined whether SD was independently and with VRFs interactively associated with prospective cognitive decline i.e., incident aMCI diagnosis, in clinically normal older adults. Our results indicate that SD was associated with an increased hazard risk of an incident aMCI. More importantly and novel is the finding that having reported SD at baseline led to 9–12 months significantly shorter time-to an incident aMCI diagnosis at a UDS follow-up, compared with not having reported SD at baseline. In addition, SD and FHS-CVD risk score were each associated with an incident aMCI when entered together into a single model. These findings underscore the importance of both sleep and vascular risk as contributors to cognitive decline in clinically normal older adults. The interaction between SD and the FHS-CVD risk score in association with prospective cognitive decline was synergistic. Stratified results showed a dose-response finding such that participants with SD and in the highest and middle tertiles of the FHS-CVD risk score were significantly more likely to develop incident aMCI during UDS follow-up, compared with participants without SD in the lowest FHS-CVD risk score tertile. Notably, the synergistic effect of SD and the FHS-CVD risk score remained strongly associated with prospective cognitive decline after adjusting for commonly used AD biomarkers, including levels of CSF-Aβ, CSF-Tau, CSF-Ptau, and hippocampal volume, and approximated the effects of these commonly used imaging biomarkers, thus suggesting their possible use as complementing the existing AD pathology markers in assessing the risk of prospective cognitive decline.

Our results show that SD is associated with an increased hazard risk of an incident aMCI diagnosis, which is in line with results from previous prospective studies examining whether self-reported sleep problems in individuals who were not demented at baseline are early independent markers of risk for developing AD or dementia over time (Sterniczuk et al., 2013; Virta et al., 2013). Metaanalysis has shown that multiple markers of SD are linked to an increased risk for all-cause dementia in older adults (Xu et al., 2020). SDs are also associated with AD pathology, in cognitively normal, late middle-aged, and older adults including Aβ and tau aggregation and regional brain atrophy (Spira et al., 2013; Branger et al., 2016; Lucey et al., 2019). The prevailing thought regarding the relationship between SD and AD is that of a bidirectional relationship (Wang and Holtzman, 2020). Our study supports the notion that SDs may precede clinical AD onset. Possible mechanistic pathways linking sleep as a risk factor for AD include the diurnal nature of the sleep-wake cycle and its effects on extracellular metabolism of Aβ and tau in the CSF and in brain interstitial fluid (ISF) as these fluctuate diurnally, with soluble Aβ levels higher during wakefulness, and lower during sleep (Kang et al., 2009). Studies in humans have demonstrated a 25–30% increase in soluble Aβ in CSF *via* increased overnight Aβ production in acute sleep-deprived individuals relative to sleeping controls (Lucey et al., 2018). In addition, Holth et al. (2019) recently showed that ISF tau also fluctuates diurnally with sleep deprivation increasing tau levels in human CSF and mouse brain ISF. Acceleration of the spreading of tau protein aggregates was seen in specific brain networks because of chronic sleep deprivation in a tau-seeding model (Holth et al., 2019). The role of sleep in amyloid pathogenesis is bolstered further with sleep deprivation studies in humans and mice showing that overnight sleep deprivation in healthy young adults significantly increased morning Aβ38, Aβ40, and Aβ42 levels in the CSF by 25–30% relative to a night of normal sleep (Lucey et al., 2018). Other possible mechanisms linking sleep as a risk factor for AD include reduced SWA (Varga et al., 2016), impaired glymphatic clearance during sleep (Xie et al., 2013), and chronic inflammation (Irwin and Vitiello, 2019).

Our results showing SD predicting a shorter progression time-to-aMCI are novel and in line with our work in patients with cognitive normal obstructive sleep apnea (OSA), and the most common is sleep disorder in the elderly. In these studies, cognitively normal OSA+ participants progressed to MCI 6–18 months earlier than OSA- participants did, depending on their amyloid and tau burden (Osorio et al., 2015; Bubu et al., 2020). In addition, we have shown that cognitively normal OSA+ subjects experience a faster annual increase in florbetapir uptake and decrease in CSF Aβ42 levels, and also increases in CSF T-tau and P-tau compared with OSA- participants (Bubu et al., 2019). More importantly, SD and FHS-CVD risk score were each associated with incident aMCI when entered together into a single model and further showed a synergistic interaction between these two factors in promoting cognitive decline, even when the model included commonly used established AD biomarkers. Our FHS-CVD risk score finding is in line with a recent study demonstrating FHS-CVD risk score associations with prospective cognitive decline (Rabin et al., 2018). VRFs are associated with lower FDG-PET (Langbaum et al., 2012), more cerebrovascular disease (CVD) (Bangen et al., 2015), higher cerebral Aβ burden (Langbaum et al., 2012; Gottesman et al., 2017), and higher tau burden (Langbaum et al., 2012; Gottesman et al., 2017), and they act synergistically with Aβ burden to promote cognitive decline (Rabin et al., 2018). The combined impact of Aβ burden and increased WMH have generally been additive on cognition (Marchant et al., 2013), thus possibly suggesting that the FHS-CVD risk score may detect other features of vascular burden not reflected by infarcts and/or WMH (Rabin et al., 2018). Possible mechanisms linking VRFs to AD risk include atherosclerosis-induced brain hypoperfusion and its impact on neurodegeneration, formation of neuritic plaques in both the hippocampus and neocortex, and hippocampal neurofibrillary tangles (Petrovitch et al., 2000). VRFs can lead to disruption of the endothelium and the blood–brain barrier and induce cerebral amyloidosis affecting cholinergic neurotransmission, which plays a critical role in normal cognition, particularly in the domains of attention, emotion, and memory (Román and Kalaria, 2006). There is also evidence that vascular protective factors may attenuate pathological processes that ultimately modify the risk of AD (Scarmeas et al., 2009).

The observed synergy between SD and FHS-CVD risk score in our study is novel and clinically relevant since a substantial number of sleep-disturbed patients have co-occurring VRFs. Possible synergistic mechanisms may include impaired cardiovascular autonomic regulation by SD (Legramante and Galante, 2005), increased sympathetic activation and oxidative stress, combined induction of cerebral amyloidosis, reduced SWA (Varga et al., 2016), and chronic inflammation (Irwin and Vitiello, 2019), by both disturbed sleep and VRFs. Many cerebrovascular changes are not well visualized on MRI (Smith et al., 2012), since conventional neuroimaging captures only a portion of the total CVD burden. Moreover, in assessing AD risk, SDs, characterized by shorter sleep duration and poor sleep quality in community-dwelling older adults are detectable using validated sleep questionnaires (Spira et al., 2013). Therefore, both sleep and VRFs may represent clinical non-invasive complements to the more invasive methods of assessing cognitive decline risk.

### Limitations

This study has its limitations; as such, results from this study are best interpreted within the context of the study sample. The NACC UDS sample consists of a convenience sampling of patients and research participants at academic ADRCs. Recruitment patterns may vary across the 33 centers. Our population included clinically normal participants at baseline followed for an incident MCI/aMCI diagnosis during a UDS follow-up. Fortunately, ADRCs employ the standardization of the clinical consensus diagnosis across the centers. The NACC database consists of mostly a white sample as such, this may affect generalizability of our findings. It is likely that sleep problems and cardiovascular outcomes are causally related; however, we ensured that there was no temporal ordering of both SD and cardio VRFs as they were obtained at baseline. As our covariates were selected *a priori*, we did not adjust for possible treatments for cardiovascular risks or disorders. However, any sort of treatment would have resulted in an attenuation of our risk estimates. The utilization of the NPI-Q to measure SD is a limitation as its inclusion in NACC UDS was because of its validity in obtaining neuropsychiatric and behavioral symptoms/syndromes’ data associated with dementia (Cummings et al., 1994). The NPI-Q sleep item conflates three separate sleep components including nocturnal awakenings, waking up too early in the morning, and excessive daytime napping behavior into one. While we argue that a positive answer to any of the three components reflects poor nighttime sleep or the presence of a sleep disorder, it cannot differentiate between possibilities (e.g., insomnia vs. hypersomnia vs. circadian rhythm abnormality), does not capture any information about difficulties in sleep initiation, which seems to be one of the most important aspects of sleep-related problems in later life that has implications for health, and negative answers may still be elicited in individuals with OSA. Notably, the NPI-Q has been validated in patients with AD (Cummings et al., 1994). Moreover, studies exist that have utilized this measure in assessing SDs as a risk factor in the elderly and in patients with AD (Bliwise et al., 2011). More importantly, the direction of the findings is in line with the existing literature suggesting SD as a risk factor for AD (Spira et al., 2013; Branger et al., 2016; Lucey et al., 2019). Another limitation is that the UDS had no data on household sleeping arrangements, thereby making it impossible to know whether some informants misreported SD, especially if they did not sleep in the same bedroom as the patients. Notably less than half of the informants lived with the participant, thus depending on the informant characteristic (e.g., coresidence, relationship with the patient—spouse, children, friend, paid caregiver), the response to this item is subject to varying degrees of reporting bias, which may include both over- and underreporting, especially given that the item may be conflated with neuropsychiatric problems. However, if this occurred, it would most likely have resulted in an aggregate underreporting of SD that would have resulted in an attenuation of our risk estimates. Furthermore, informants’ reliability was extremely high and we controlled for informants’ characteristics in our models. Another limitation is concerning CSF and MRI measures. To guard against differences in CSF assays and MRI imaging, we utilized CSF data measured using the same assay method (i.e., ELISA) and MRI data from same high-resolution T1-weighted anatomical imaging scans. We also tried to account for center differences statistical wise by including the center-ID in the adjusted models. Lastly, many NACC UDS participants have some level of advanced education, and may therefore have some level of cognitive reserve. However, we expect this to have attenuated our estimates. Our results suggest that even with some level of reserve, vascular risk can interact with sleep to accelerate cognitive decline.

## Conclusion

In summary, our results suggest that SD and vascular risk are strongly associated with prospective cognitive decline, both alone and in synergism beyond commonly used biofluid and imaging AD biomarkers, in cognitively normal older adults. We, therefore, propose a framework for additional research. Examples include: (a) using objective measures to examine whether sleep-vascular risk synergism related to cognitive decline is independent of Aβ and tau pathology and (b) determine specific sleep and vascular disorders’ effects on slow wave and rapid eye movement sleep and their mediating role in increasing Aβ and tau accumulation. More importantly, these less invasive clinical measures of sleep and vascular risk may actually serve as possible complements of the more invasive biofluid and imaging biomarkers in assessing the risk of prospective cognitive decline in cognitively normal older adults. The relevance of these findings is bolstered by epidemiological studies suggesting that an approximate combined 20–23% [15% for sleep problems/disorders (Bubu et al., 2017) and 5–8% for hypertension (Barnes and Yaffe, 2011)] of AD may be prevented should interventions be implemented to reduce sleep problems/disorder, and hypertension.

## Data Availability Statement

Publicly available datasets were analyzed in this study. This data can be found here: https://naccdata.org/requesting-data/nacc-data.

## Ethics Statement

Ethical review and approval was not required for the study on human participants in accordance with the local legislation and institutional requirements. Written informed consent for participation was not required for this study in accordance with the national legislation and the institutional requirements.

## Author Contributions

OB, EW, OU-B, AKM, and RO contributed to the conception and design of the study. OB, EW, OU-B, ADT, AKM, JC, and JB contributed to the acquisition and analysis of data. OB, EW, OU-B, SK, ADT, JC, AEM, AP, KK, JB, ZO, AN, ART, ML, DR, IA, GO, GJ-L, AV, AVM, and RO drafted a significant portion of the manuscript, tables, or figures. All authors contributed to the article and approved the submitted version.

## Conflict of Interest

The authors declare that the research was conducted in the absence of any commercial or financial relationships that could be construed as a potential conflict of interest.

## Publisher’s Note

All claims expressed in this article are solely those of the authors and do not necessarily represent those of their affiliated organizations, or those of the publisher, the editors and the reviewers. Any product that may be evaluated in this article, or claim that may be made by its manufacturer, is not guaranteed or endorsed by the publisher.

## Footnotes

1
https://www.alz.washington.edu

2
http://surfer.nmr.mgh.harvard.edu/

3
https://www.alz.washington.edu
